# Enhancing Efficiency in Coal-Fired Boilers Using a New Predictive Control Method for Key Parameters

**DOI:** 10.3390/s26010330

**Published:** 2026-01-04

**Authors:** Qinwu Li, Libin Yu, Tingyu Liu, Lianming Li, Yangshu Lin, Tao Wang, Chao Yang, Lijie Wang, Weiguo Weng, Chenghang Zheng, Xiang Gao

**Affiliations:** 1 State Key Laboratory of Clean Energy Utilization, Institute of Carbon Neutrality, State Environmental Protection Engineering Center for Coal-Fired Air Pollution Control, Zhejiang University, Hangzhou 310027, China; 2 Zhejiang HOPE Environmental Protection Engineering Co., Ltd., Hangzhou 310013, China; 3 Zhejiang Materials Industry Group Corporation (ZJMI) Environmental Energy Co., Ltd., Hangzhou 310003, China; 4 Jiaxing Research Institute, Zhejiang University, Jiaxing 314000, China

**Keywords:** coal-fired boiler, predictive control method, operational parameter optimization, Transformer, GRU

## Abstract

In the context of carbon neutrality, the large-scale integration of renewable energy sources has led to frequent load changes in coal-fired boilers. These fluctuations cause key operational parameters to deviate significantly from their design values, undermining combustion stability and reducing operational efficiency. To address this issue, we introduce a novel predictive control method to enhance the control precision of key parameters under complex variable-load conditions, which integrates a coupled predictive model and real-time optimization. The predictive model is based on a coupled Transformer-gated recurrent unit (GRU) architecture, which demonstrates strong adaptability to load fluctuations and achieves high prediction accuracy, with a mean absolute error of 0.095% and a coefficient of determination of 0.966 for oxygen content (OC); 0.0163 kPa and 0.987 for bed pressure (BP); and 0.300 °C and 0.927 for main steam temperature (MST). These results represent substantial improvements over lone implementations of GRU, LSTM, and Transformer models. Based on these multi-step predictions, a WOA-based real-time optimization strategy determines coordinated adjustments of secondary fan frequency, slag discharger frequency, and desuperheating water valves before deviations occur. Field validation on a 300 t/h boiler over a representative 24 h load cycle shows that the method reduces fluctuations in OC, BP, and MST by 62.07%, 50.95%, and 40.43%, respectively, relative to the original control method. By suppressing parameter variability and maintaining key parameters near operational targets, the method enhances boiler thermal efficiency and steam quality. Based on the performance gain measured during the typical operating day, the corresponding annual gain is estimated at ~1.77%, with an associated CO_2_ reduction exceeding 6846 t.

## 1. Introduction

Motivated by global climate change and sustainable development objectives, countries worldwide have actively promoted the large-scale deployment of renewable energy, including wind and solar photovoltaic power [1,2]. However, large-scale grid integration of these renewable energy sources has significantly increased operational volatility and uncertainties in power systems [3,4]. To mitigate these fluctuations and maintain grid stability, coal-fired boilers must perform rapid and frequent load adjustments [5,6], resulting in key operational parameters deviating significantly from their design values. Various studies [7,8,9] have demonstrated that these deviations reduce boiler efficiency, increase fuel consumption and compromise operational stability. Therefore, enhancing the control precision of key parameters under variable-load conditions and suppressing their deviations from design values is essential for ensuring safe and efficient boiler operation.

The combustion process in coal-fired boilers is inherently complex, as it exhibits substantial nonlinearity, significant time delays, and strong coupling among multiple operational parameters. These characteristics render the accurate regulation of operational parameters highly challenging, particularly under varying operating conditions. In the control of boiler parameters, cascaded PID (proportional-integral-derivative) control is widely applied because of its simplicity and ease of implementation. However, it has the drawbacks of slow response time and a limited ability to reject disturbances.

To address these limitations, previous studies and engineering practices have verified the feasibility and effectiveness of applying model predictive control (MPC) methods to coal-fired boilers [10]. Huang et al. [11] used a constrained MPC method to optimize a boiler desulfurization system, leading to improved desulfurization efficiency. Zlatkovikj et al. [12] proposed a feed-forward MPC, which achieved lower deviations in fluidized bed temperature, boiler heat load, and fluidized bed temperature compared to other controllers, contributing to more stable combustion and energy output. Similarly, Yang et al. [13] developed a two-time-scale nonlinear MPC framework to enhance the operational flexibility and control performance of coal-fired power plants integrated with post-combustion carbon capture systems. The effectiveness of MPC is determined by the prediction accuracy of its models, which are typically derived from state-space, transfer functions, derived as step/impulse response models via system identification. However, under large load disturbances and non-stationary operating conditions in practical boiler systems, the assumption of linear predictive models becomes unreliable, leading to model-plant mismatch and time-varying delays that deteriorate control performance.

Recent studies have increasingly explored data-driven deep learning (DL) methods for combustion process modeling, which eliminate the need for detailed representations of physical and chemical processes [14]. MA et al. [15] introduced a novel model that integrates a stacked target-enhanced autoencoder with a long short-term memory (LSTM) based on an attention mechanism to predict the oxygen content. Li et al. [16] developed a predictive model that integrates multiple neural networks, significantly improving the prediction accuracy of oxygen content and furnace temperature. Tan et al. [17] proposed a multistep steam temperature predictive model based on LSTM, which significantly improved prediction accuracy under complex operating conditions. Ye et al. [18] proposed an integrated Transformer and CNN models to mitigate the impact of operational delays in boiler systems and predict key parameters of circulating fluidized bed boilers, and the results demonstrated that Transformer architecture can achieve superior performance in learning from sequential boiler data. An increasing body of research has substantiated the superior prediction accuracy of DL models for key boiler parameters, laying a foundation for integrating data-driven modeling with advanced control frameworks.

Building upon these advances, several studies have investigated integrated DL methods within MPC frameworks, demonstrating improved control performance in variable-load conditions [19,20]. In addition, data-driven models combined with intelligent optimization algorithms have been explored to enhance the regulation of key parameters [21,22]. For instance, Wang et al. [23] integrated genetic algorithms (GA) with MPC to develop a multivariable boiler control strategy, aiming to reduce steady-state energy consumption while improving response speed and disturbance rejection capability. Moreover, Alitasb et al. [24] proposed a radial basis function neural network (RBFNN)-based MPC. Compared with conventional state-space model-based MPC, this method achieved faster settling times, smaller overshoot, and superior dynamic response in the control of temperature, pressure, and drum water level. The above methods have demonstrated good performance in numerical simulations; however, their application to online closed-loop control in actual coal-fired boilers remains challenging.

Despite the progress in data-driven modeling and MPC for coal-fired boilers, several key challenges remain. Existing studies (i) mostly focus on single-parameter modeling, with limited work on multi-parameter systems under load disturbances, and (ii) are evaluated mainly in simulations, with few studies validating their real-time performance in actual boilers.

In this study, we propose a predictive control method for key operational parameters of coal-fired boilers, focusing on accurate control of the oxygen content in flue gas (OC), the bed pressure (BP), and the main steam temperature (MST). A coupled predictive model based on the Transformer integrated with GRU is developed to enable real-time forecasting and enhance the prediction accuracy for key operational parameters. Building on this predictive model, a real-time optimal control strategy employing the whale optimization algorithm (WOA) is designed to adaptively adjust the secondary air flow, slag discharger frequency, and openings of the desuperheater valves. The proposed method is then implemented and validated on a 300 t/h coal-fired boiler. It allows coordinated adjustments of key operational parameters in advance of deviations, thereby improving boiler thermal efficiency and steam quality.

## 2. Coal-Fired Boiler System and Data Preprocessing

### 2.1. Introduction to Boiler System

**Figure 1:**
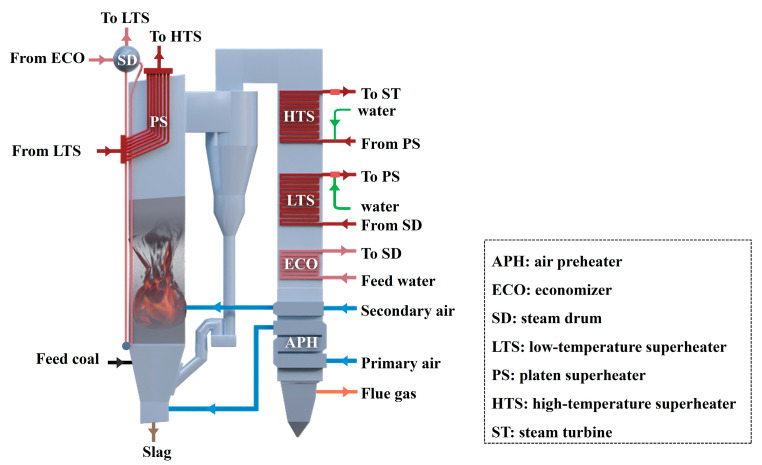
Schematic diagram of a boiler system.

As shown in Figure 1, coal particles are fed into the furnace through the feeding system and suspended in the combustion chamber by the high-pressure primary air supplied through nozzles at the bottom of the bed. To ensure complete combustion of the coal particles, an appropriate amount of oxygen is supplied via secondary air nozzles arranged along the furnace walls. A mixture of partially combusted coal and ash rises with the flue gas to the upper region of the furnace and is then directed into a cyclone separator, where the heavier particles are separated from the flue gas and recirculated to the furnace. This is performed to maintain the fluidization and combustion cycles. Fine particles are discharged with the flue gas as fly ash, while the large particles that cannot be elutriated are discharged as bottom slag. The slag discharge system regulates the discharge rate to maintain the stability of the gas–solid two-phase flow within the bed. Meanwhile, the feedwater is heated and evaporated into saturated steam in the water-wall tubes, and enters the steam drum. The steam then exits the steam drum and flows sequentially through the low-temperature superheater, first-stage desuperheater, platen superheater, second-stage desuperheater, and high-temperature superheater, finally entering the steam turbine.

### 2.2. Problem Definition

The coal-fired boiler is a multi-input, multi-output system characterized by considerable control delays, strong nonlinearities, and high coupling among relevant variables. Boiler load adjustments through variations in the coal feed rate inherently led to significant fluctuations in operational parameters, resulting in deviations of the parameters from their design values. Such deviations directly result in decreased boiler operational efficiency and heightened operational safety risks. A detailed analysis of the boiler system indicates that the OC [25], BP [26], and MST [27] are key operational parameters that characterize combustion performance and operational stability under variable-load conditions.

OC is affected by primary air, secondary air, and coal feed rate, reflecting the balance between air supply and fuel input. When the air supply is insufficient, local oxygen deficiency limits the oxidation of volatiles and char, leading to elevated CO concentration, higher unburned carbon in fly ash and bottom slag, and an increased risk of slagging. Conversely, excessive air supply raises OC and enhances carbon burnout efficiency, but increases flow and temperature of flue gas, resulting in higher heat loss. Therefore, maintaining OC at the design value is crucial to ensure complete combustion while minimizing thermal losses.

BP is affected by coal feed rate and slag discharge, reflecting the gas–solid flow state and the inventory of circulating solids in the furnace, and directly determining the fuel residence time, mixing intensity, and heat release distribution. An excessively low BP indicates insufficient solids holdup and weak particle circulation, causing fuel particles to be rapidly carried away from the combustion zone before complete burnout, thus reducing combustion efficiency and destabilizing the temperature field. In contrast, an excessively high BP corresponds to excessive solids accumulation, which increases flow resistance, raises fan power consumption, and may deteriorate fluidization quality, leading to local agglomeration and uneven heat release. Maintaining a stable BP helps ensure adequate fuel residence time and uniform gas–solid contact, promoting safe and efficient combustion.

MST is affected by furnace combustion and desuperheating water flow rate, reflecting the quality of the steam. Excessively high MST may impose thermal stress on heat exchange surfaces, increasing the risk of material degradation, while excessively low MST results in reduced steam quality, which can degrade turbine efficiency and reliability.

Importantly, OC, BP, and MST exhibit strong coupling during the combustion and heat transfer processes. Variations in OC modify fuel burnout efficiency and combustion intensity, which in turn affect bed hydrodynamics and solids circulation, thereby leading to variations in BP. Conversely, variations in BP modify solids holdup and gas–solid mixing, thereby altering the local oxidizing environment and OC. These interactions further affect combustion completeness and the heat release, rendering MST susceptible to significant fluctuations. Under variable-load operation, demand-driven adjustments of fuel feed, air supply, and slag discharge simultaneously affect these key parameters. This strong interdependence makes OC, BP, and MST highly susceptible to fluctuations, leading to their frequent deviation from design values.

We establish a predictive control framework for a coal-fired boiler, which involves the prediction of three key operational parameters and generation of the corresponding control commands for their respective manipulating variables. Specifically, OC is mainly regulated by the frequency of the secondary air fan (SFF); BP is controlled primarily through the frequency of the slag discharge (SDF); and MST is chiefly adjusted via the opening of the desuperheating water valve (DHV).

**Figure 2:**
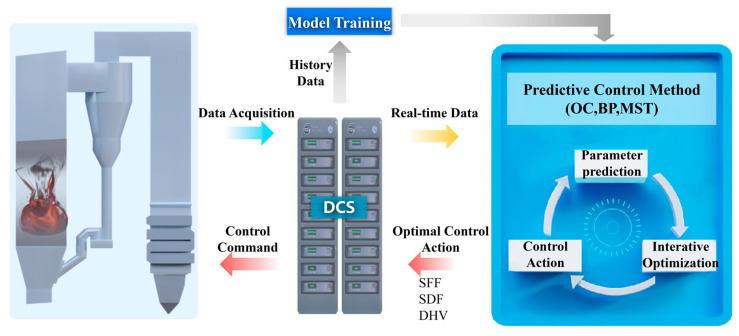
Deployment architecture of the predictive control system for boiler operational parameters.

As illustrated in Figure 2, the predictive model is deployed on an industrial personal computer (IPC). It communicates with the distributed control system (DCS) via the Modbus TCP protocol, and performs real-time acquisition of boiler operating data and closed-loop delivery of control commands.

### 2.3. Data Preprocessing

Operational data of the 300 t/h coal-fired boilers were collected via the DCS during routine industrial operation, covering a 3-month period with a 5 s sampling interval. The dataset reflects typical load fluctuations and operational conditions, providing a representative basis for modeling the dynamic relationships between key boiler parameters. It includes a wide range of measured variables, summarized in Table 1, which are relevant for characterizing boiler performance. The dataset was split into training, validation, and test subsets in a 6:2:2 ratio for model training and evaluation.

During the data preprocessing phase, the raw dataset was cleaned to remove outliers, sensor noise, and invalid readings. A sliding-window median filter was applied to fill the missing values and maintain temporal continuity.

To capture time-delay effects, a sliding-window scheme is used to construct historical input sequences $H_{t}=\{s_{t-L+1},s_{t-L+2},\ldots ,s_{t}\}$*,* where $s_{i}\in R^{m}$ denotes the sample at the ith time step with m features. *L* is the window length, and $H_{t}$ represents the historical sequence up to time *t*. The corresponding output is $Y_{t}=\{y_{t+1},y_{t+2},\ldots ,y_{t+T}\}$, where $y_{t+\tau }\in R^{m}$ denotes the key operational parameters at time step t+τ, and T is the prediction horizon.

For the three key parameters of the coal-fired boiler, the dataset is further partitioned into three subsets:

Common variable set: $C=\{c_{1},c_{2},\ldots ,c_{p}\}$, representing variables that simultaneously affect the three parameters, such as coal feed rate, main steam flow, total air flow, and feedwater flow, which reflect the overall thermal input and load conditions.

Manipulated variable set: $K=\left\{k_{1}^{j},k_{2}^{j},\ldots ,k_{q}^{j}\right\},\;j\in \{\mathrm{OC},\;\mathrm{BP},\;\mathrm{MST}\}$, where each subset consists of the manipulated variables of the corresponding key parameter. For example, the secondary air flow rate and fan frequency for OC, the operating frequency of the slag discharge system for BP, and the flow rates and valve openings of the desuperheating water for MST.

Derived variable set: $D=\{d_{1},d_{2},\ldots ,d_{r}\}$, which is computed from the original operational data. The construction of derived parameters varies depending on the key parameters, including the ratio of primary to secondary air flow, the deviation of the slag discharger frequency from its rated frequency, and the temperature difference between the intermediate superheater point and the main steam.

## 3. Coupled Modeling and Real-Time Control

### 3.1. Operational Parameter Predictive Model

**Figure 3:**
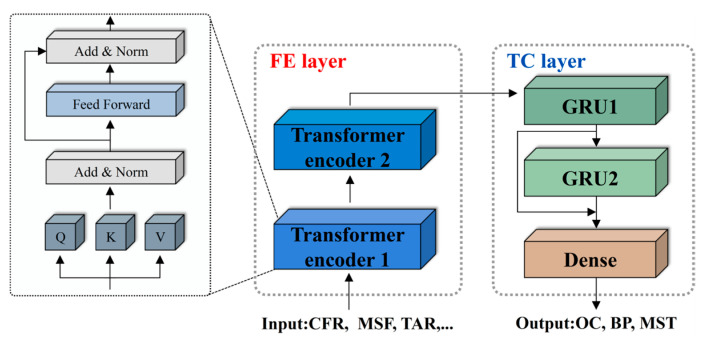
Structure diagram of the predictive model.

A unified coupled modeling framework that integrates the Transformer with the GRU is developed to predict three key operational parameters of boilers. This hybrid design leverages the complementary strengths of the two components: the Transformer extracts global and inter-variable coupling relationships, while the GRU captures localized temporal dependencies and short-term dynamics. As illustrated in Figure 3, the proposed predictive model is organized into two functional layers:

(1) Feature extraction (FE) layer: composed of stacked Transformer encoder modules, responsible for extracting nonlinear relationships among multi-dimensional input features and capturing long-range dependencies within the sequences.

(2) Temporal capture (TC) layer: consisting of residual-connected GRUs, responsible for modeling transient behavior and evolving temporal trends of the parameters.

The model takes the input sequences of all parameters (defined in Section 2.3), processes them through the FE and TC layers, and outputs the predicted future values of OC, BP, and MST. By using a shared input space and joint processing, the model captures the coupling and cross-influences among the three parameters, rather than treating them independently.

As the first stage of the predictive framework, the FE layer is designed to capture high-dimensional interactions among boiler operational variables. This layer is implemented using stacked Transformer encoders, which are particularly effective at modeling nonlinear dependencies and long-range relationships in multivariate time series. The core strength of the Transformer encoder lies in its self-attention mechanism [28,29], which quantifies the contribution of each input variable by computing the relationships among key operational variables. To achieve this, the input feature matrix $X\in R^{T\times d}$ is first projected into three distinct spaces through linear transformations, resulting in the query (*Q*), key (*K*), and value (*V*) representations:

(1) $$Q=XW^{Q},\;K=XW^{K},\;V=XW^{V}$$

where $W^{Q}$, $W^{K}$, $W^{V}$ are learnable weight matrices. The attention output is obtained as:

(2) $$Attention\left(Q,K,V\right)=softmax\left(\frac{QK^{T}}{\sqrt{d_{k}}}\right)V$$

where $d_{k}$ denotes the dimension of the key vectors. The dot product of the input features generates attention scores, which represent their influence on the key operational parameters at the current time step.

To enhance the representation power, multi-head attention is adopted. Multiple attention heads focus on different subspaces of the feature representation in parallel. This specific mechanism is formalized in Equations (3) and (4):

(3) $$MultiHead\left(Q,K,V\right)=Concat\left({head}_{1},\ldots ,{head}_{h}\right)W^{O}$$

(4) $${head}_{i}=Attention\left(QW_{i}^{Q},KW_{i}^{K},VW_{i}^{V}\right)$$

where Concat(.) denotes the row-wise concatenation of all attention heads, $W^{O}$ denotes the output weight matrix. This design enables the model to simultaneously capture diverse coupling patterns among operational parameters.

While the Transformer effectively captures global dependencies among operational parameters, it is less capable of modeling short-term temporal variations. To address this limitation, the TC layer is constructed using two GRUs. Each GRU regulates information flow through the update gate and the reset gate, enabling the model to selectively retain past information and integrate new input. A residual connection between the two GRUs further enhances feature fusion across layers, improving the model’s representational capacity and flexibility. The GRU update equations are defined as follows:

(5) $$\begin{array}{c} \begin{array}{c} \gamma_{t}=\sigma \left(W_{\gamma h}h_{t-1}+W_{\gamma x}x_{t}+b_{r}\right)\\ {\tilde{h}}_{t}=\tanh\left(W_{\tilde{h}h}h_{t-1}*\gamma_{t}+W_{\tilde{h}x}x_{t}+b_{h}\right) \end{array}\\ \begin{array}{c} z_{t}=\sigma \left(W_{zh}h_{t-1}+W_{zx}x_{t}+b_{z}\right)\\ h_{t}=\left(1-z_{t}\right)*h_{t-1}+z_{t}*\tilde{h_{t}} \end{array} \end{array}\}$$

where σ denotes the sigmoid activation function, $h_{t-1}$ is the hidden state from the previous time step, and $x_{t}$ represents the input vector; $W_{\gamma h}$, $W_{\gamma x}$, $W_{\tilde{h}h}$, $W_{\tilde{h}x}$, $W_{zh}$, and $W_{zx}$ denote the corresponding weight matrices, and $b_{r}$, $b_{h}$ and $b_{z}$ denote the bias vectors. This mechanism enables the GRU to effectively capture short-term states and dynamic trends of key operational parameters, while maintaining relatively low computational complexity.

### 3.2. Real-Time Optimization Control

A real-time optimization control strategy is developed for the key parameters based on the predictive model. For each key parameter, the optimal adjustment of the corresponding manipulated variable for the next time step is determined via the WOA [30]. The objective function for each parameter y is defined as follows:

(6) $$f=\sum_{i=1}^{m}{\left(y_{p}\left(t+i\right)-y_{d}\right)}^{2}+C\sum_{j=1}^{n}{\left(∆u\left(t+j\right)\right)}^{2}$$

where m and n represent the prediction and control horizons, respectively, $y_{p}$ denotes the predicted result, $y_{d}$ denotes the design value, ∆u(t+j) represents the adjustment of the corresponding manipulated variable, and C the penalty coefficient for the control actions. By minimizing this objective function, the strategy determines the optimal control actions that reduce deviations of key parameters from their design values, and avoids excessive variations in the manipulated variables.

**Figure 4:**
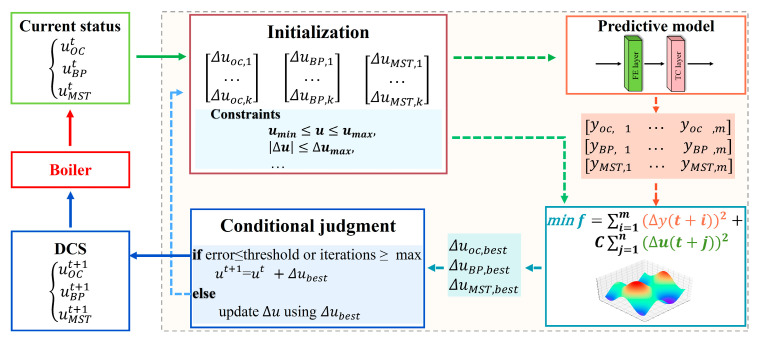
Schematic diagram of the real-time optimization control framework.

The manipulated variables in this study correspond to the key boiler actuators: $u_{OC}$ represents the frequency of the secondary air fan, $u_{BP}$ denotes the frequency of the slag discharge, and $u_{MST}$ represents the opening of the desuperheating water valve. The manipulated variables are subject to the following constraints:

(7) $$\begin{array}{c} u_{min}\leq u\left(t+j\right)\leq u_{max}\\ \left|∆u\left(t+j\right)\right|=\left|u\left(t+j\right)-u\left(t+j-1\right)\right|\leq ∆u_{max}\\ y_{low}\leq y_{p}\left(t+i\right)\leq y_{high} \end{array}\}$$

where u(t+j) denotes a manipulated variable of the boiler system, $u_{min}$ and $u_{max}$ are its lower and upper bounds, respectively, $∆u_{max}$ the maximum change in the manipulated variable, $y_{low}$ and $y_{high}$ the safe lower and upper limits of the three key parameters, respectively. A schematic diagram of the real-time optimization control framework is shown in Figure 4.

The WOA is used to determine the optimal manipulation commands for the key operational parameters to minimize the objective function. This algorithm replicates the predation strategy of humpback whales, in particular their distinctive spiral bubble-net feeding technique [30,31,32].

The implementation details of the WOA are shown in Table 2. The WOA dynamically selects the position update strategy at each iteration based on the probability p and the coefficient vector A, enhancing its global exploration capability and improving its ability to escape local optima.

When p<0.5 and |A|<1, the encircling mechanism is employed, allowing search agents to perform a focused local search around the current best solution. This gradually narrows the scope of the encirclement of the whale group, allowing them to surround the prey, as follows:

(8) $$X\left(t+1\right)=X^{*}\left(t\right)-A\cdot \left|CX^{*}\left(t\right)-X\left(t\right)\right|$$

where X(t) denotes the positional vector of the current search agent, $X^{*}\left(t\right)$ denotes the positional vector of the best search agent at the current iteration, A and C represent coefficient vectors, which are determined as:

(9) A=2αr−α

(10) C=2r

(11) $$\alpha =2-\frac{2t}{MaxIter}$$

here r represents a randomly generated vector in [0, 1], α denotes the convergence factor, and MaxIter denotes the maximum number of iterations.

When p<0.5 and |A|≥1, the randomization mechanism is utilized to enhance global exploration. In this case, the search agent moves toward a randomly selected agent ($X_{rand}\left(t\right)$), allowing the algorithm to escape potential local optima and explore previously unvisited regions of the search space, as follows:

(12) $$X\left(t+1\right)=X_{rand}\left(t\right)-A\cdot \left|CX_{rand}\left(t\right)-X\left(t\right)\right|$$

When p≥0.5, the spiral mechanism is employed, simulating the hunting behavior of whales as they approach prey along a spiral trajectory. This mechanism promotes local exploitation around the current best solution, as follows:

(13) $$X\left(t+1\right)=X^{*}\left(t\right)-\left|X^{*}\left(t\right)-X\left(t\right)\right|*e^{bL}\cos\left(2\pi L\right)$$

where b denotes a fixed constant, and L represents a random value within [−1, 1].

The predictive control framework operates with a 5 s control interval, consistent with the sampling interval. The Transformer–GRU model is trained offline and only used for online forward prediction, while the WOA optimizes the manipulated variables, including secondary fan frequency, slag discharger frequency, and desuperheating water valve openings. The computational load of each control cycle remains well within the 5 s interval, ensuring real-time feasibility.

To ensure operational reliability, a state-based convergence monitoring and disturbance-free switching mechanism is implemented. If the optimization converges within a control cycle, the corresponding control commands are applied; otherwise, a non-convergence signal triggers an automatic fallback to the original control model in the DCS, ensuring safe and continuous boiler operation until convergence is restored.

### 3.3. Implementation Details and Evaluation Protocol

Model training and evaluation were conducted on a computer equipped with an NVIDIA GeForce RTX 4080 GPU. The experiments were implemented in Python 3.9 with PyTorch 1.13. To assess the prediction accuracy, several baseline models—GRU, LSTM, and Transformer—were implemented and trained under identical conditions. All predictive model training was performed using the Adam optimizer with a fixed learning rate of 0.001 and a batch size of 512. The prediction performance was quantified by the mean absolute error (MAE) and the coefficient of determination (R^2^).

To evaluate the effectiveness of the proposed predictive control method, the results were compared with the existing original control method (cascade control), and the control performance was evaluated using the standard deviation (*SD*):

(14) $$SD=\sqrt{\frac{\sum_{i=1}^{n}{\left(y_{i}-y_{d}\right)}^{2}}{n}}$$

where n represents the number of samples, $y_{i}$ denotes the measured value.

## 4. Results and Discussion

### 4.1. Predictive Performance Comparison of Models

**Figure 5:**
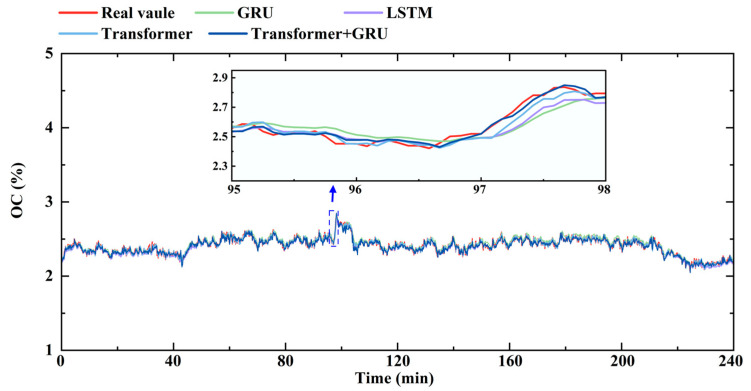
Comparison of the predicted and measured OC for different models.

**Figure 6:**
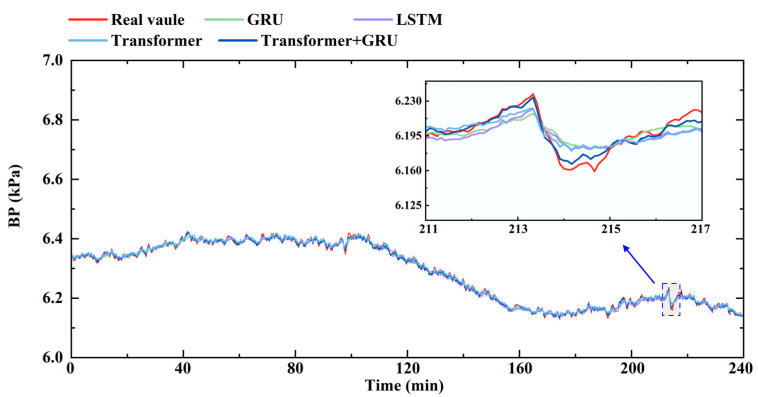
Comparison of the predicted and measured BP for the different models.

**Figure 7:**
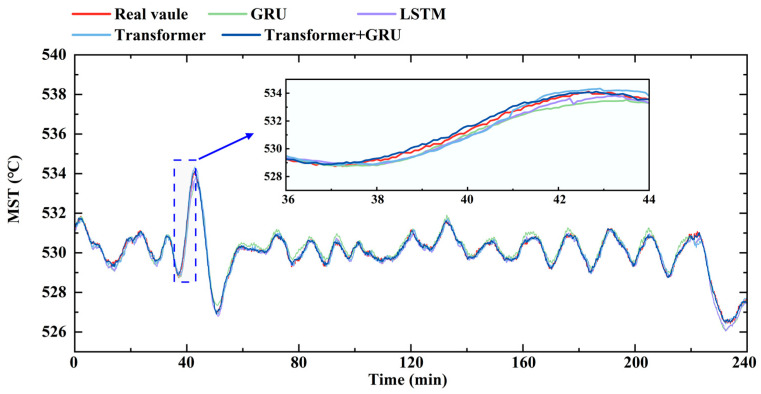
Comparison of the predicted and measured MST for the different models.

A continuous 240 min data segment for each operational parameter was selected from the test set, collected from an actual 300 t/h coal-fired boiler in operation, was selected to compare the predictive performance of the different models, as illustrated in Figure 5, Figure 6 and Figure 7. We can see that OC demonstrates an intermediate response speed, but is affected by the probe’s downstream location in the tail flue, leading to a longer delay of approximately 75 s, and accordingly, the prediction at the 15th timestep was selected. In contrast, BP directly reflects the gas–solid two-phase flow in the furnace and responds rapidly, with a typical delay of approximately 35 s, corresponding to a 7-step prediction horizon. MST exhibits significant thermal inertia due to the heat transfer dynamics within the furnace and superheater system, resulting in an approximate delay of 3 min.

As illustrated in Figure 5, all models reasonably capture the variational trend of OC; however, their predictive performance diverges under load disturbances. During such fluctuations, the oxygen concentration responds to variations in fuel feed and airflow, reflecting the complex dynamics of the combustion process. In these conditions, the GRU and LSTM models exhibit relatively limited accuracy and fail to fully represent these variations, whereas the Transformer and Transformer–GRU models more effectively track the dynamic trends. Among them, the Transformer–GRU achieves the lowest MAE (0.095%) and highest R^2^ (0.966), exhibiting the best predictive performance and demonstrating effectiveness in extracting both short-term dynamics and long-term dependencies.

BP is influenced by fuel feed rate and airflow. During load fluctuations, rapid changes in primary air flow and fuel feed rate can lead to significant disturbances in bed void fraction and solid distribution, resulting in pronounced BP variations. The predictive model needs to accurately forecast these BP fluctuations resulting from changes in fuel feed and airflow under load variations. As shown in Figure 6, the Transformer–GRU model achieves the best predictive performance for BP, with a MAE of 0.0163 kPa and an R^2^ of 0.987, outperforming the GRU (MAE = 0.0199 kPa, R^2^ = 0.965), LSTM (MAE = 0.0187 kPa, R^2^ = 0.971), and the Transformer (MAE = 0.0185 kPa, R^2^ = 0.971). These results indicate that the Transformer–GRU model can more accurately capture rapid BP fluctuations and reflect the underlying combustion dynamics.

MST is primarily affected by the heat absorbed from the furnace and the steam flow through the superheater. Under load fluctuations, variations in combustion intensity and uneven heat release can lead to significant MST fluctuations. As shown in Figure 7, the GRU and LSTM models exhibit relatively higher prediction errors when MST undergoes large fluctuations, failing to fully capture the dynamic variations in MST under load fluctuations. The Transformer model, leveraging its attention mechanism, better tracks the trend, achieving an MAE of 0.327 °C and an R^2^ of 0.905. The Transformer–GRU model further improves predictive performance, achieving an MAE of 0.300 °C and an R^2^ of 0.927, indicating its effectiveness in accurately capturing MST fluctuations.

The MAE and R^2^ of all models for each operational parameter are presented in Table 3.

### 4.2. Control Effectiveness of Typical Operational Conditions

Prevailing safety regulations and operational guidelines for boilers make it difficult to deploy and validate all comparative predictive models in an actual operational environment. Therefore, the coupled Transformer–GRU model, which exhibited the best predictive performance, was selected for online deployment and control strategy validation, with the predictive control method replacing the original method to regulate three key operational parameters. The predictive results from this model are used in a WOA-based real-time optimization to determine optimal control increments and suppress fluctuations of key operational parameters.

(1) Steady-load operating conditions

**Figure 8:**
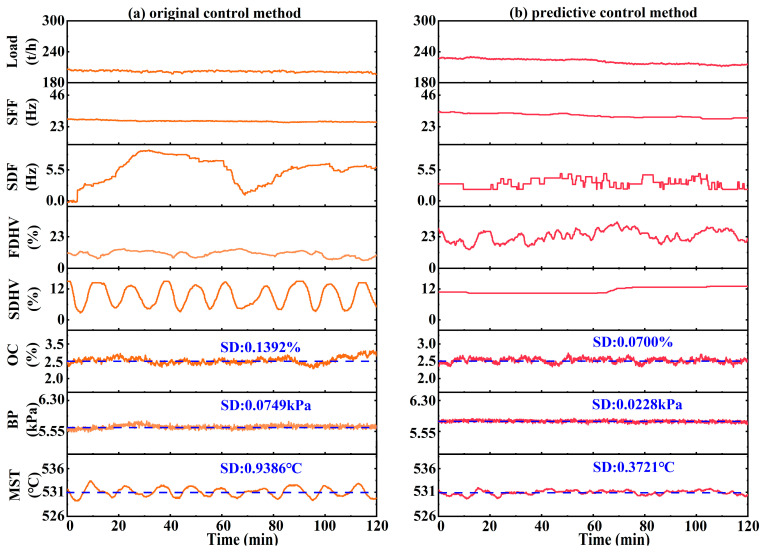
Comparison of control performance for key parameters under steady-load conditions: (**a**) original control method; (**b**) proposed predictive control method.

The control performances of OC, BP, and MST under steady-load operating conditions were comparatively analyzed, as illustrated in Figure 8.

Under the original control method (Figure 8a), the three key parameters exhibited relatively large fluctuations around their respective design values. Specifically, OC fluctuated within a wide range of 2.29–2.81%, with an SD of 0.1392%. BP deviated between −0.13 and 0.16 kPa, resulting in an SD of 0.0749 kPa. For MST, the temperature ranged from 528.75 °C to 533.17 °C, with an SD of 0.9386 °C. These results indicate that the original control method cannot promptly compensate for disturbances, leading to wider parameter variations and lower control precision.

In contrast, the predictive control method (Figure 8b) achieved markedly superior performance by integrating a predictive model with an optimization framework, enabling more frequent and proactive adjustments. OC followed its design value more closely, with its fluctuation range reduced to 2.34–2.73% and an SD decreased to 0.0700%. The BP deviations narrowed to the range of −0.10 kPa to 0.09 kPa, with the SD significantly reduced to 0.0228 kPa. Similarly, the MST variation was effectively suppressed, with the range narrowed to 529.62 °C–531.97 °C, and the SD significantly decreased to 0.3721 °C.

(2) Rapid load change operating conditions

**Figure 9:**
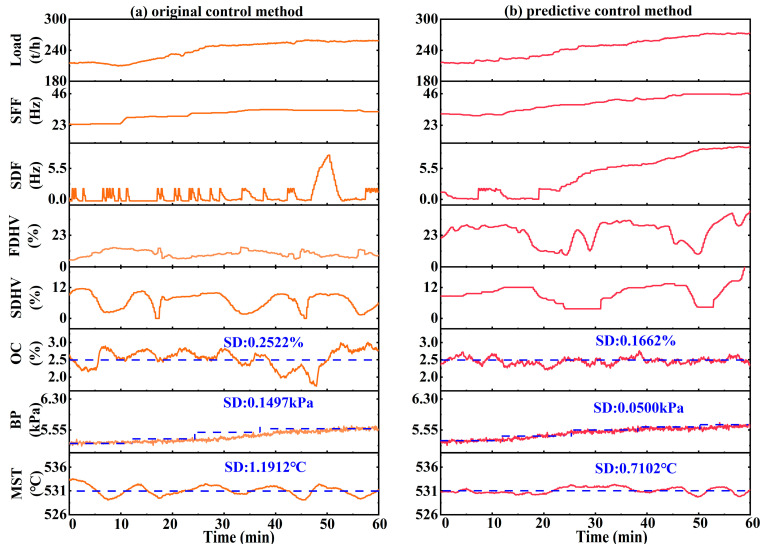
Comparison of control performance for key parameters under rapid load conditions: (**a**) original control method; (**b**) proposed predictive control method.

The control performance of OC, BP, and MST under rapid load change operating conditions was comparatively analyzed, as illustrated in Figure 9.

Under the original control method (Figure 9a), the three parameters exhibited significant fluctuations and delayed responses to load disturbances. Specifically, relative to its design value, OC ranged from 1.73% to 2.96%, with an SD of 0.2522%. And due to the lag in secondary air flow adjustments, OC exhibited overshoot and could not effectively track its design value. BP stability was also affected, as evidenced by an SD of 0.1497 kPa, indicating pronounced instability in the gas–solid two-phase flow within the bed, and slow dynamic responses under load transitions. For MST, the system exhibited delayed responses followed by overshoot, resulting in temperature deviations between −1.91 °C and 2.50 °C, with an SD of 1.1912 °C. These results demonstrate that the original control method lacks robustness under rapid load changes, leading to poor rejection of disturbances.

As shown in Figure 9b, the predictive control method achieved significantly better regulation. Relative to its design value, OC was confined within a narrower range of 2.21–2.76%, with the SD markedly reduced to 0.1662%, effectively suppressing overshoot and enhancing tracking accuracy. The control performance of BP was also significantly improved, with the SD reduced to 0.0500 kPa, indicating a substantial improvement in the stability of the gas–solid two-phase flow and a faster dynamic response. Similarly, MST deviations were effectively mitigated, as the variation range narrowed to −1.26 °C to 1.34 °C and the SD decreased to 0.7102 °C.

### 4.3. Evaluation of Operational Efficiency

**Figure 10:**
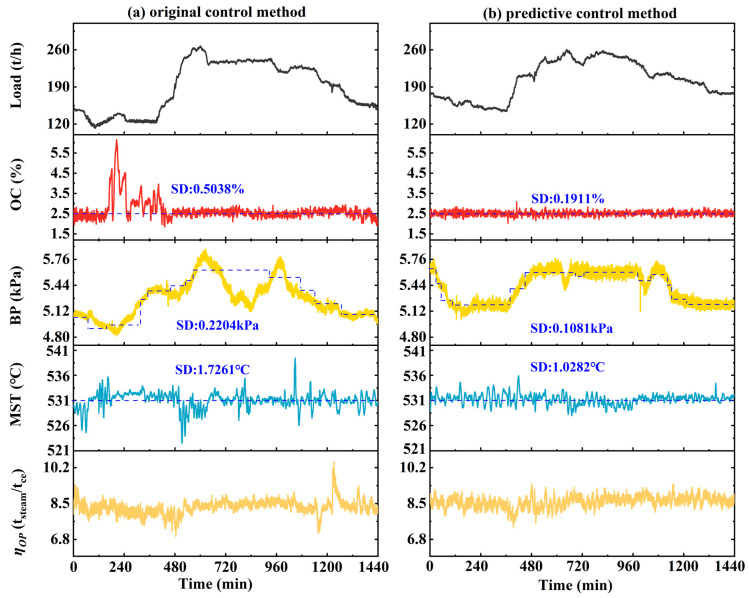
Comparison of operating data on a typical day.

A comparative analysis between the predictive control method and the original control method was conducted for a typical day, which encompasses a complete 24 h load variation cycle; this is considered representative of a boiler’s actual operating conditions. As illustrated in Figure 10, under the proposed predictive control method, the fluctuation amplitudes and standard deviations of OC, BP, and MST are all significantly lower than under the original control method.

For OC, the original control method resulted SD of 0.5038%, with higher nighttime levels observed due to the difficulties in regulating secondary air distribution under ultra-low secondary air flow conditions. In contrast, the predictive control method reduced the SD to 0.1911%, representing a 62.07% reduction. For BP, the design value dynamically varies with the boiler load. Under the original control method, the bed pressure was unable to effectively follow this dynamic design values, exhibiting an SD of 0.2204 kPa. In contrast, the predictive control method proactively adjusted the bed material discharge, maintaining a BP close to the design value, with the SD reduced to 0.1081 kPa; this corresponds to a reduction of 50.95%. The control performance of MST also showed similar improvement, with its SD decreased by 40.43% as compared to the original method. Moreover, the predictive control method exhibited more stable instantaneous specific steam generation compared with the original control method.

By effectively suppressing fluctuations in OC, BP, and MST, and holding these key parameters close to their design values, the predictive control method reduces unnecessary energy losses and improves fuel utilization. To quantify this effect, the boiler operational efficiency was evaluated by calculating the ratio of the total steam production to the total standard coal equivalent (tce) consumed during a typical day, hereafter denoted as $\eta_{OP}$. On this typical day, $\eta_{OP}$ increased from 8.4288 tsteam/tce under the original control method to 8.5777 t_steam_/tce under the predictive control method, corresponding to a relative improvement of 1.77%. Based on this observed improvement and the annual operating records of the boiler, the corresponding annual reductions in coal consumption and CO_2_ emissions were estimated to be approximately 2471 tce and 6846 t, respectively, with an associated cost saving of about RMB 2.47 million. These values should be interpreted as indicative estimates, as actual long-term performance may vary with fuel properties, operating schedules, and operating conditions.

Table 4 presents a comparison of the operational indicators and economic benefits of the two methods.

### 4.4. Limitations

The present study has several limitations. First, the proposed approach was validated on a typical day, and longer-term continuous operation is required to further assess and refine the model. Second, the method has been validated on a single industrial boiler, and its generalizability to boilers with different configurations, fuel types, or operational strategies remains to be evaluated. Finally, the proposed method currently focuses on boiler-side control, and future work could incorporate the turbine system to expand the range of controllable variables and further enhance overall operational performance.

## 5. Conclusions

We found that the Transformer–GRU model demonstrates high accuracy in multi-step forecasting, with an MAE of 0.095% and R^2^ of 0.966 for OC; 0.0163 kPa and 0.987 for BP; and 0.300 °C and 0.927 for MST. The results demonstrate that the proposed coupled Transformer–GRU model outperforms GRU, LSTM, and Transformer models in terms of prediction accuracy.

The whale optimization algorithm was used to achieve real-time optimization and adjustment of key parameters based on the results of the predictive model and operational constraints. Compared to the original control method, the fluctuations in OC, BP, and MST were reduced by 62.07%, 50.95%, and 40.43%, respectively. These findings demonstrate that the proposed method can accurately regulate key operational parameters under variable-load conditions.

The proposed predictive control method substantially suppresses deviations in key parameters, ensures more complete fuel combustion, and increases fuel utilization by 1.77% during a representative operating day, thereby enhancing combustion stability and operational efficiency under complex, rapidly changing load conditions. By extrapolating this typical-day performance, the corresponding annual reduction in coal consumption is estimated at approximately 2471 tce, with an associated CO_2_ reduction of about 6846 t.

## Figures and Tables

**Table 1:** Boiler operating data.

Variable Name	Units
Coal feed rate, CFR	t/h
Main steam flow, MSF	t/h
Total air flow, TAR	t/h
Feed water flow, FWL	t/h
Oxygen content, OC	%
Primary air flow, PAF	m^3^/h
Secondary air flow, SAF	m^3^/h
Furnace differential pressure, FDP	kPa
Bed pressure, BP	kPa
Secondary fan frequency, SFF	Hz
Slag discharge frequency, SDF	Hz
Flue gas temperature, FGT	°C
Superheater tube wall temperature, SWT	°C
Feed water temperature, FWT	°C
Superheater inlet steam temperature, SIST	°C
Superheater outlet steam temperature, SOST	°C
Main steam temperature, MST	°C
First-stage desuperheating water flow, FDHWF	t/h
Second-stage desuperheating water flow, SDHWF	t/h
First-stage desuperheater valve opening, FDHV	%
Second-stage desuperheater valve opening, SDHV	%

**Table 2:** The implementation details of the WOA.

Step	Description
Step 1	Initialize the whale population and define the fitness function (Equation (6))
Step 2	Evaluate the fitness of each whale and identify the current best solution
Step 3	Update the algorithm parameters *p* and A; then, update the whale positions according to one of Equation (8), Equation (12), or Equation (13)
Step 4	Recalculate the fitness values for the updated positions and update the global best solution if improved
Step 5	Repeat Steps 3–4 until the maximum number of iterations is reached; then output the optimal control adjustment command

**Table 3:** MAE and R2 for the different predictive models.

Performance Metrics	Model	OC	BP	MST
**MAE**	GRU	0.111	0.0199	0.379
	LSTM	0.107	0.0187	0.321
	Transformer	0.101	0.0185	0.327
	Transformer–GRU	0.095	0.0163	0.300
**R^2^**	GRU	0.936	0.965	0.890
	LSTM	0.939	0.971	0.891
	Transformer	0.947	0.971	0.905
	Transformer–GRU	0.966	0.987	0.927

**Table 4:** Comparison of operational indicators and economic benefits.

Indicator	Original Control Method	Predictive Control Method	Improvement	Relative
OC fluctuation (SD, %)	0.5038	0.1911	−0.3127	−62.07%
BP fluctuation (SD, kPa)	0.2204	0.1081	−0.1123	−50.95%
MST fluctuation (SD, °C)	1.7261	1.0282	−0.6979	−40.43%
$\eta_{OP}$ (t_steam_/tce)	8.4288	8.5777	+0.1489	+1.77%
Annual steam (t)	1,200,000	1,200,000	—	—
Coal consumption (tce)	142,369.02	139,897.64	−2471.38	−1.74%
Coal price (RMB/tce)	1000	1000	—	—
Annual fuel cost (RMB)	142,369,020	139,897,640	−2471.380	−1.74%
CO_2_ emissions (t)	394,362.19	387,516.46	−6845.73	−1.74%

## Data Availability

The data presented in this study are available on request from the corresponding author due to privacy.
